# Influence of the amino acid residues at 70 in M protein of porcine reproductive and respiratory syndrome virus on viral neutralization susceptibility to the serum antibody

**DOI:** 10.1186/s12985-016-0505-7

**Published:** 2016-03-22

**Authors:** Baochao Fan, Xing Liu, Juan Bai, Tingjie Zhang, Qiaoya Zhang, Ping Jiang

**Affiliations:** Key Laboratory of Animal Diseases Diagnostic and Immunology, Ministry of Agriculture, College of Veterinary Medicine, Nanjing Agricultural University, Nanjing, 210095 P.R. China; Jiangsu Co-innovation Center for Prevention and Control of Important Animal Infectious Diseases and Zoonoses, Yangzhou, China

**Keywords:** PRRSV, 70, M protein, Neutralizing antibody

## Abstract

**Background:**

Porcine reproductive and respiratory syndrome virus (PRRSV) is mainly responsible for the significant economic losses in pig industry in the world. The adaptive immune responses of the host act as an important source of selective pressure in the evolutionary process of the virus. In the previous study, we confirmed that the amino acid (aa) residues at 102 and 104 sites in GP5 played an important role in escaping from the neutralizing antibodies (NAbs) against highly pathogenic PRRSV (HP-PRRSV). In this study, we further analyzed the aa mutants affecting neutralization susceptibility of NAbs in other structure proteins in NAbs resistant variants.

**Methods:**

Based on the different aa residues of the structural proteins between the resistant virus BB20s and the parent virus BB, 12 recombinant PRRSV strains containing these aa residue substitutions were constructed using reverse genetic techniques. The neutralizing antibody (NA) titers of the recombinant strains were tested on MARC-145 and porcine alveolar macrophages (PAMs). And the NAbs binding abilities of parent and rescued viruses were tested by using ELISA method.

**Results:**

By using the neutralization assay, it was revealed that the NA titer of N4 serum with rBB/Ms was significantly lower than that with rBB. Meanwhile, NA titer of the serum with rBB20s/M was significantly higher than that with rBB20s. The ELISA binding results showed that rBB/Ms had higher binding inability to N4 than did rBB. And alignment of M protein revealed that the variant aa residue lysine (K) at 70 was also existed in field type 2 and vaccine PRRSV strains.

**Conclusions:**

The aa residue at 70 in M protein of PRRSV played an important role in regulating neutralization susceptibility to the porcine serum NAbs. It may be helpful for monitoring the antigen variant strains in the field and developing new vaccine against PRRSV in the future.

**Electronic supplementary material:**

The online version of this article (doi:10.1186/s12985-016-0505-7) contains supplementary material, which is available to authorized users.

## Background

Since described in USA in 1987, porcine reproductive and respiratory syndrome (PRRS) has become one of the most important diseases in pigs, leading to significant economic losses in swine industry worldwide [1–3]. The causative agent, porcine reproductive and respiratory syndrome virus (PRRSV), was identified in 1991 in the Netherlands [4] and 1992 in the United States [5]. In 2006, a HP-PRRSV strain with discontinuous 30 aa residues deletion in nsp2 protein associated with porcine high fever syndrome was reported in China, and overwhelmed swine industries in China and Vietnam [6–8]. PRRSV genome is approximately 15.4 kb in length and has at least 10 open reading frames (ORFs) [9]. Among them, ORF2a, ORF2b, ORF3-7, and ORF5a encode eight structural proteins: a small, non-glycosylated E protein, five glycosylated membrane proteins (GP2-GP5 and GP5a), a non-glycosylated membrane protein (M), and the nucleocapsid protein (N) [10–13].

The GP2, GP3, and GP4 interact with each other to form a multiprotein complex that is dispensable for viral particle formation and important for viral infectivity [14–18]. GP4 of the European genotype PRRSV strain Lelystad contains a a highly immunogenic epitope located at the region spanning aa 57–68, which induces neutralizing antibodies [19–22]. However, the locations of these epitopes have not been mapped completely [23–25]. The M protein encoded by ORF6 is an unglycosylated membrane protein of 18–19 kDa [26–28], and is important in virus assembly and budding [29]. The M is linked to GP5 as heterodimers via a disulfide bond in the N-terminal ectodomains [27, 30]. The M protein is a key target for PRRSV neutralization [31]. Co-expression of GP5 and M protein as heterodimers significantly improves the potency of PRRSV DNA vaccination [32]. Anti-M mAbs have been described, but the neutralizing epitopes in M gene have not yet been identified [23, 31].

The adaptive immune response of the host will act as an important source of selective pressure in the evolutionary process of the virus [33, 34]. Costers et al. [35, 36] isolated the antibody escape variants from the vaccinated pigs, and reveled that vaccination-induced or infection-induced intermediate levels of neutralizing antibodies might be considered as an important driving force in PRRSV evolution. In addition, a large dataset analysis defined two hypervariable regions and several positive aa sites in GP5 under selective evolutionary pressure, which may globally favor the survival of the fittest variants to infect and persist within the host [37]. In the previous study, we obtained the HP-PRRSV NAbs resistant strains and determined that the 102 and 104 aa sites in GP5 played an important role in escaping from the NAbs against HP-PRRSV. But the sequencing results showed that the structure proteins GP2, GP3, GP4 and M of the resistant strains also contained some aa substitutions compared with the parent virus [38]. In this study, we further analyzed the remaining aa mutants affecting neutralization susceptibility of antibody in the different structure proteins by using reverse genetic techniques and virus neutralization assay. It was found that the aa at site 70 in M protein was another aa site, which affected the neutralization susceptibility of NAbs.

## Results

### Rescue of recombinant viruses and identification of the antibody binding sites

**Fig. 1:**
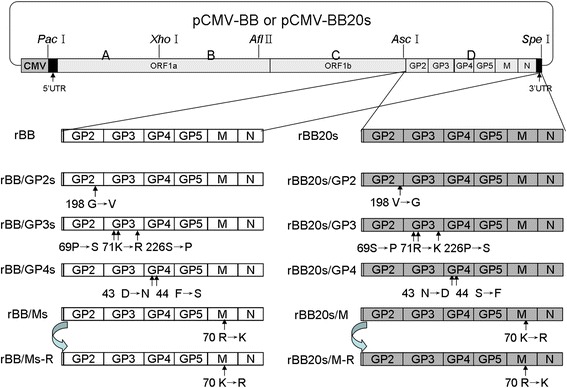
The construction strategy of infectious cDNA clones of the recombinant PRRSVs using BB and BB20s strains. ^a^ The rBB/Ms-R and rBB20s/M-R were two revertant viruses and constructed from the full-length infectious cDNA clone pCMV-BB/Ms and pCMV-BB20s/M by using the site-directed mutagenesis, respectively

To elucidate the role of the aa mutations in the structural proteins of BB20s in the virus’s resistant ability, five recombinant PRRSV strains, rBB, rBB/GP2s, rBB/GP3s, rBB/GP4s and rBB/Ms that contained the aa mutations in the separate protein according to BB20s were rescued from BB strain as shown in Fig. 1. In addition, in order to definite that the results were due to the aa mutants, one revertant virus rBB/Ms-R was also generated. All the recombinant viruses and the aa mutations are summarized in Table 1.

**Table 1:** The recombinant viruses used in this study and the related mutant amino acid sites

Name	aa mutant sites	Name	aa mutant sites
rBB	No	rBB20s	No
rBB/GP2s	GP2(198G/V)	rBB20s/GP2	GP2(198 V/G)
rBB/GP3s	GP3(69P/S,71 K/R,226S/P)	rBB20s/GP3	GP3(69S/P,71R/K,226P/S)
rBB/GP4s	GP4(43D/N;44 F/S)	rBB20s/GP4	GP4(43 N/D;44S/F)
rBB/Ms	M(70R/K)	rBB20s/M	M(70 K/R)
rBB/Ms-R ^a^	No	rBB20s/M-R ^b^	No

^a^ The recombinant virus rBB/Ms-R was a revertant virus from rBB/Ms and contained 70R in M. The plasmid was constructed from the full-length infectious cDNA clone pCMV-BB/Ms by using the site-directed mutagenesis described

^b^ The recombinant virus rBB20s/M-R was a revertant virus from rBB20s/M and contained 70 K in M. The plasmid was constructed from the full-length infectious cDNA clone pCMV-BB20s/M by using the site-directed mutagenesis described

**Fig. 2:**
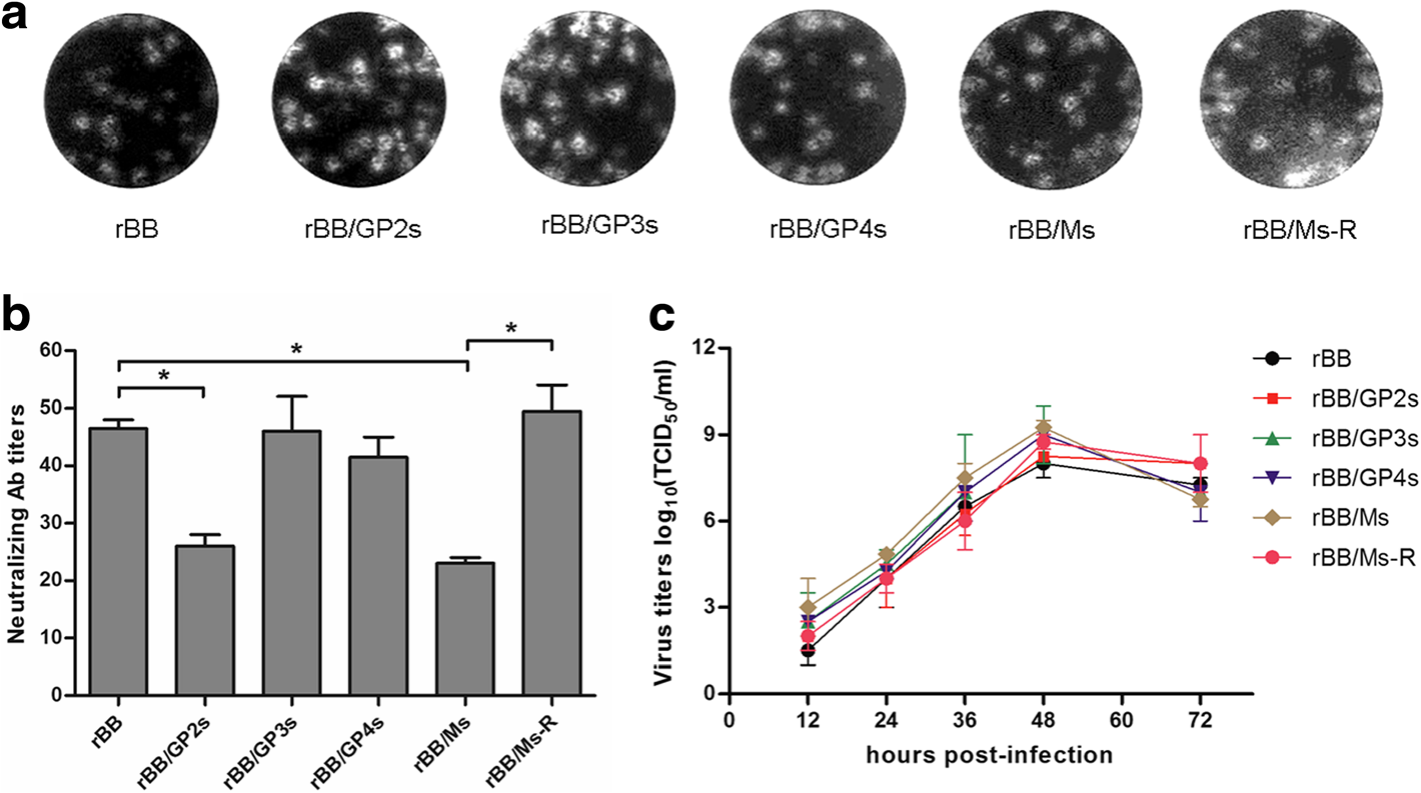
Characterization of the recombinant PRRSV strains rBB, rBB/GP2s, rBB/GP3s, rBB/GP4s, rBB/Ms and revertant virus rBB/Ms-R. **a** Plaque morphology assay. **b** Virus neutralization assays of recombinant viruses by using the N4 serum. **c** The growth kinetics. The data are represented as the means ± s.d. of three independent experiments, and significant differences are shown (*P < 0.05) by using one-way analysis of variance (ANOVA)

Plaque assay results showed that the plaques of rBB/GP2s, rBB/GP3s, rBB/GP4s and rBB/Ms were similar as those of parent strain rBB (Fig. 2a). The NA titers of the serum with rBB/GP2s and rBB/Ms were significantly lower than those with rBB (*P* < 0.05), and the NA titer of rBB/Ms was the lowest (Fig. 2b). Meanwhile, the revertant virus rBB/Ms-R had similar NA titers with rBB (Fig. 2b). Moreover, viral replication kinetic results showed that the replication capacities of rBB/GP2s, rBB/GP3s, rBB/GP4s and rBB/Ms were similar with rBB (Fig. 2c).

**Fig. 3:**
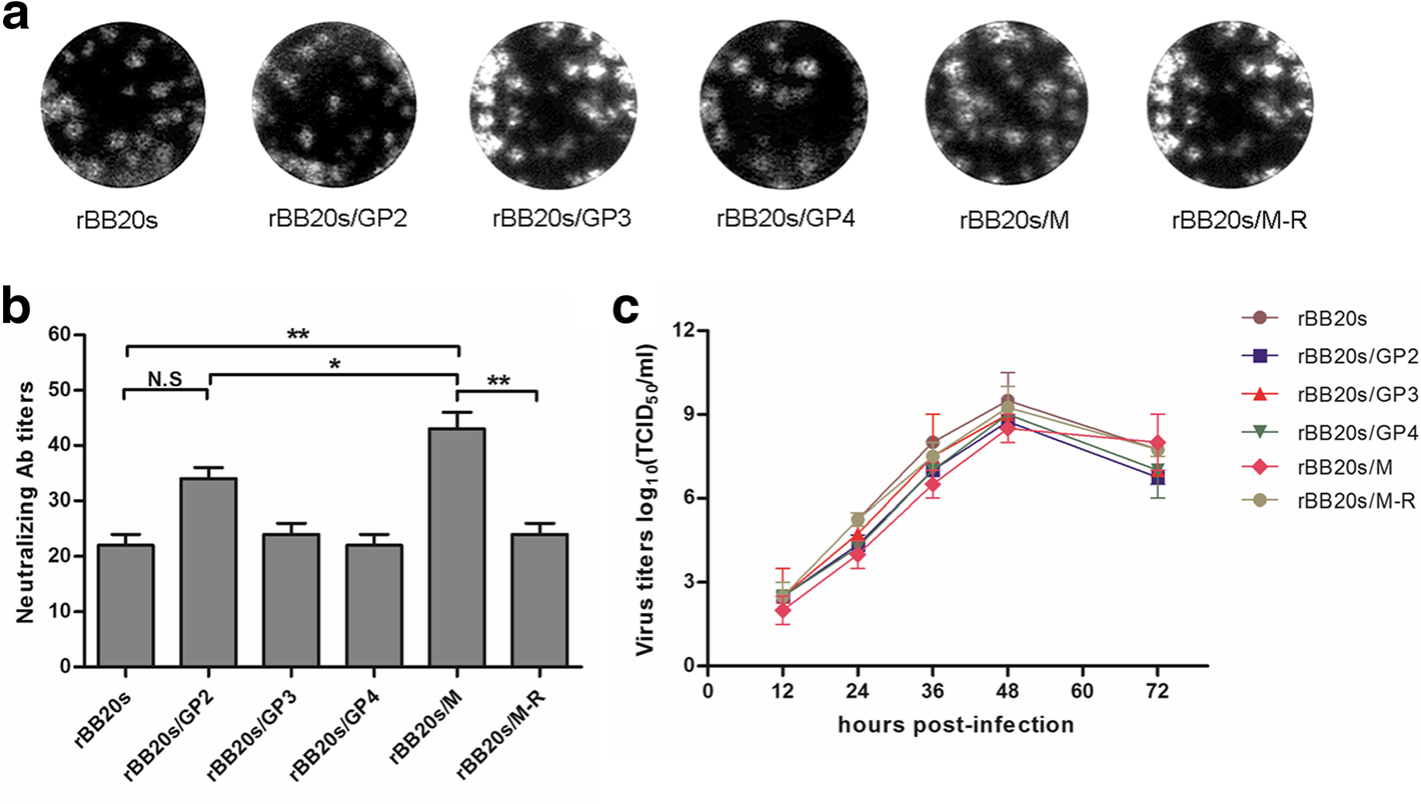
Characterization of the recombinant PRRSV strains rBB20s, rBB20s/GP2, rBB20s/GP3, rBB20s/GP4, rBB20s/M and revertant virus rBB20s/M-R. **a** Plaque morphology assay. **b** Virus neutralization assays of recombinant viruses by using the N4 serum. **c** The growth kinetics. The data are represented as the means ± s.d. of three independent experiments, and significant differences are shown (*P < 0.05 and **P < 0.01) by using one-way analysis of variance (ANOVA)

To confirm the role of the aforementioned aa mutations, a recombinant PRRSV strain, rBB20s, was rescued from plasmid pCMV-BB20s, which contained the full-length genome of BB20s. Four additional recombinant PRRSV strains, rBB20s/GP2, rBB20s/GP3, rBB20s/GP4 and rBB20s/M that contained the aa mutations in the separate protein according to BB were rescued from BB20s (Fig. 1). As shown in Fig. 3a, the plaques of the recombinant viruses were similar with each other. NA titer of N4 serum with rBB20s/M was significantly higher than that with rBB20s (*P* < 0.01). The NA titer of rBB20s/GP2 was higher than that of rBB20s, but there was no significant difference in NA titers between these two strains (*P* > 0.05) (Fig. 3b).

These data suggested that the aa residue at 70 in M played an important role in escaping from NAbs against HP-PRRSV.

### Binding analysis of resistant mutants

**Fig. 4:**
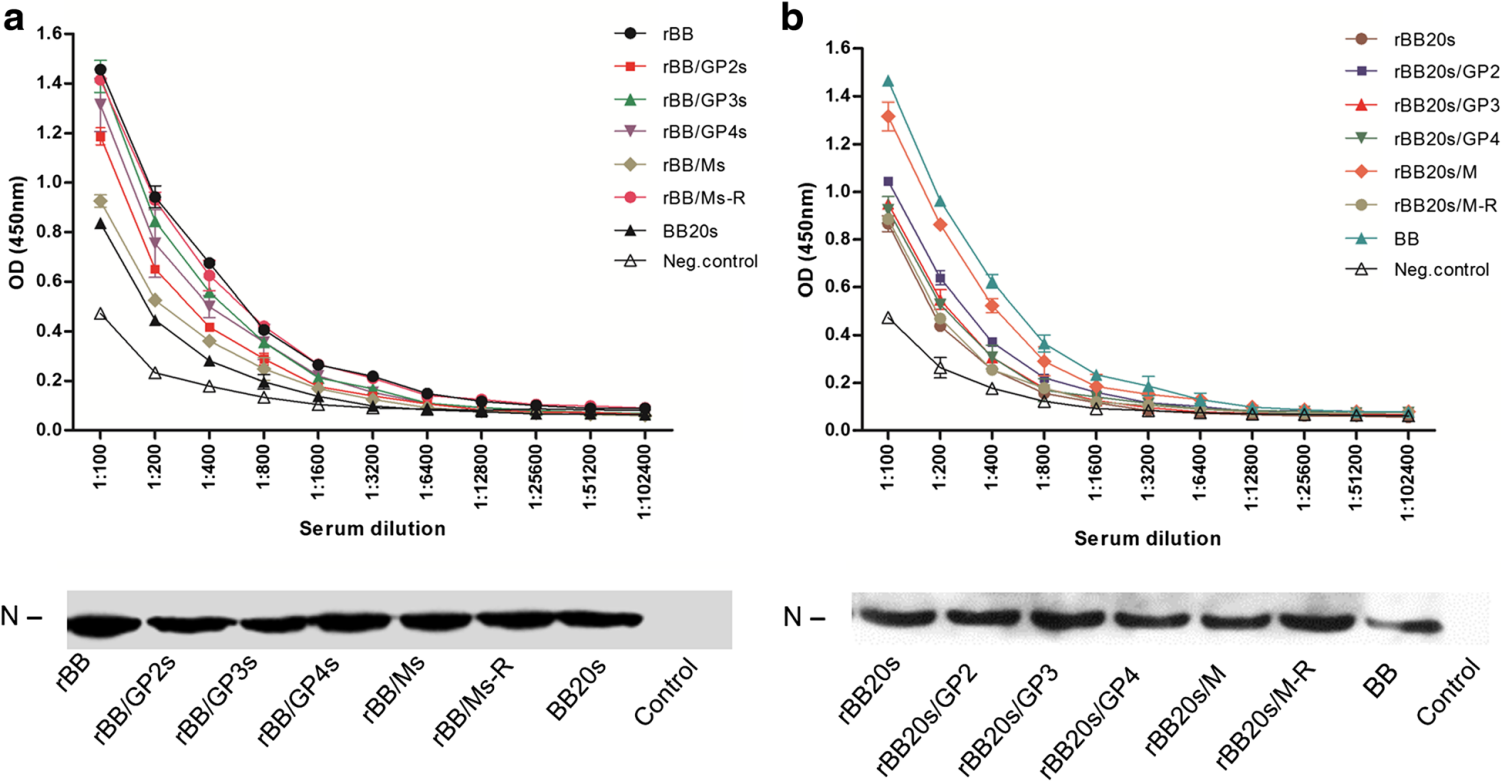
Binding analysis of the resistant and recombinant mutant strains. A binding ELISA was performed using plates coated with each of the indicated viruses in triplicate. Serial dilutions of N4 serum were added to the wells, followed by the addition of a secondary anti-pig antibody conjugated to HRP. OD values were read at 450 nm. The negative control was wells that coated with ultracentrifuged non-infected MARC-145 cell lysates. The consistent concentration of ultracentrifugal PRRSV viruses that coated on the plate were identified by using Western Blot of N proteins. **a** rBB and related mutant viruses. **b** rBB20s and related mutant viruses

ELISA binding was used to confirm that the escape ability of the virus was due to the antigenic mutations. As shown in Fig. 4, the results of western blot showed that the binding antigens in the plates that coated with the purified viruses at the same concentration almost had the same amount of N protein. Meanwhile, the ELISA results showed that the evasion virus BB20s had the lowest OD values compared with other virus groups (Fig. 4a). rBB/Ms had higher binding inability to N4 than did rBB. In addition, the remaining virus strains rBB/GP2s, rBB/GP3s and rBB/GP4s showed a binding pattern similar to that of rBB.

Figure 4b showed the results of rBB20s and the related mutant viruses. rBB20s/GP2, rBB20s/GP3 and rBB20s/sGP4 had similar OD values with their parent virus rBB20s. However, the aa mutant virus rBB20s/M significantly increased the N4 binding abilities and had similar OD values to BB.

### Resistances of the escape variants against different serum NAbs to HP-PRRSV

The NA titers of the remaining four antibody samples were measured by using the different strains obtained as above. As shown in Table 2, the NA titers of the serums with rBB/GP2s were reduced when compared those with the rBB. However, the decrease in the magnitude of the NA titers was not large. Meanwhile, the NA titers with rBB20s and rBB/Ms were significantly reduced when compared to those with the parent virus rBB. The neutralizing abilities of the serums with rBB20s/M were significantly higher than those with rBB20s. It confirmed that the aa residues at 70 in M played an important role in the binding ability of PRRSV to its NAbs.

**Table 2:** The neutralizing antibody titers of the remaining four serums against the six different PRRSV strains

Virus	Neutralizing antibody titers (Mean ± s.d.)			
	N1	N2	N3	N5
rBB	38 ± 2	34 ± 4	42 ± 5	40 ± 3
rBB20s	16 ± 4	14 ± 2	16 ± 6	20 ± 4
rBB/GP2s	28 ± 2	30 ± 3	24 ± 5	32 ± 4
rBB20s/GP2	26 ± 4	18 ± 2	26 ± 3	24 ± 3
rBB/Ms	24 ± 2	16 ± 2	24 ± 3	20 ± 1
rBB20s/M	31 ± 3	28 ± 4	34 ± 6	36 ± 2

The data are represented as the means ± s.d. of three independent experiments

### Single replication cycle virus-neutralization test of resistant variants on PAMs

**Fig. 5:**
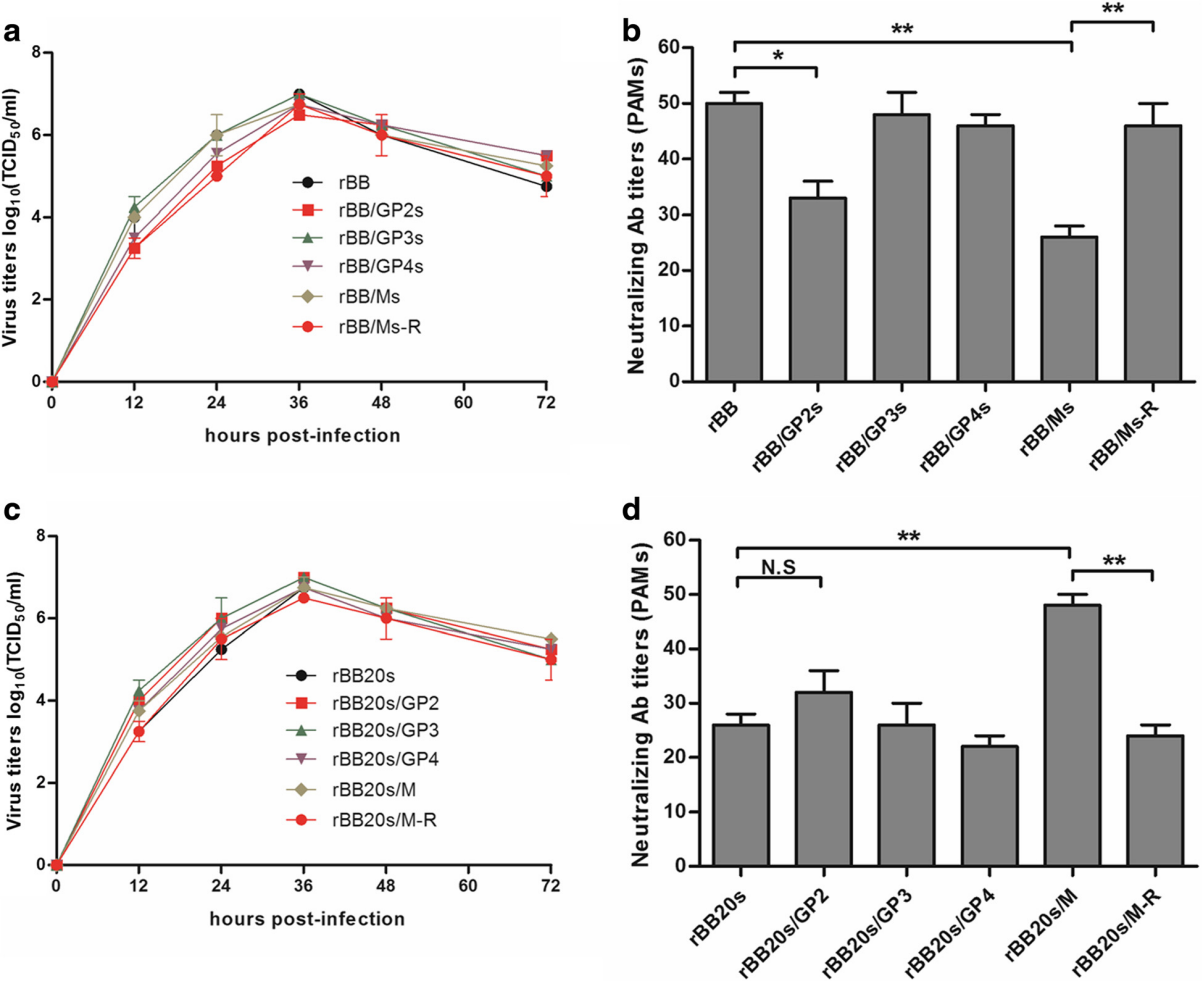
Virus-neutralization test of resistant variants on PAMs. The growth kinetics of rBB and related viruses (**a**), rBB20s and related viruses (**c**). Virus neutralization assays of rBB and related viruses (**b**), rBB20s and related viruses (**d**). The data are represented as the means ± s.d. of three independent experiments, and significant differences are shown (*P < 0.05 and **P < 0.01) by using one-way analysis of variance (ANOVA)

As shown in Fig. 5a, the replication kinetics of rBB and the related recombinant viruses on PAMs were similar. rBB/GP2s and rBB/Ms significantly reduced the NA titers of N4 on PAMs when compared with rBB (Fig. 5b). Moreover, Fig. 5c showed that the replication kinetics of rBB20s and the related viruses were similar, and the NA titer with rBB20s/M was significantly higher than that with rBB20s. The NA titers of rBB20s/GP2 slightly rose when compared with rBB20s, but the difference was not significant (Fig. 5).

### Comparison of the aa sequences of PRRSV isolates in the field

**Fig. 6:**
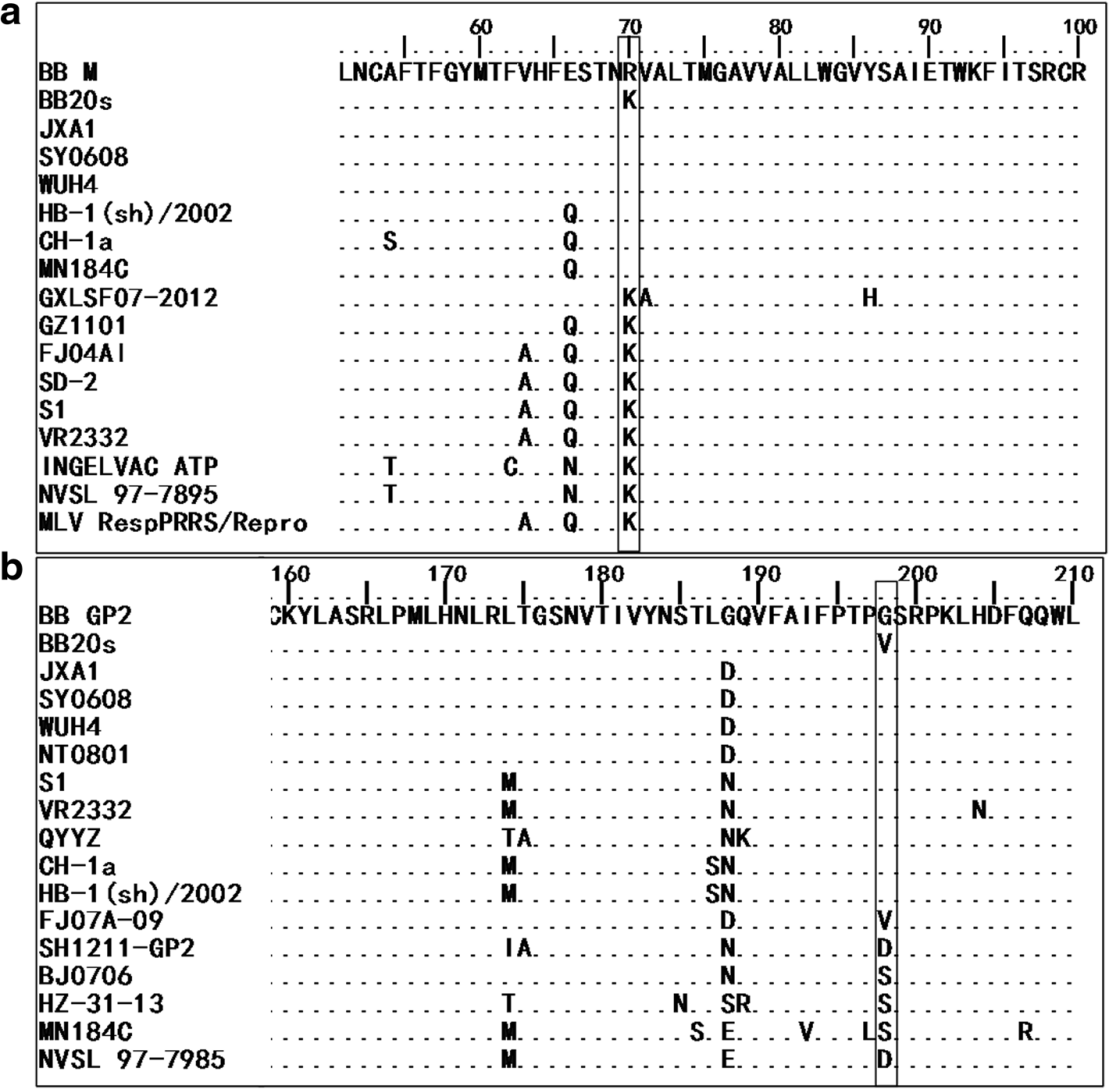
Alignment of partial M (**a**) and GP2 (**b**) aa sequences of BB20s with wild-type PRRSV isolates. The 70 aa site in M and 198 aa site in GP2 of BB20s and the same sites of the field PRRSV isolates are indicated by black boxes

In this study, the aa substitution sites in the structural proteins were acquired by immune-pressured passages in vitro. Thus, we detected whether these mutations were also existed in field PRRSV isolates. As shown in Fig. 6, some field isolates also had the same aa mutants as the resistant virus BB20s. For example, PRRSV strains S1, VR2332 and FJ1405 contained the same 70 K in the M protein as the resistant virus BB20s (Fig. 6a). It is noticeable that the vaccine strains lngelvac PRRS ATP and MLV also contained the 70 K in M protein. PRRSV strain FJ07A-09 had the same aa substitution (198 V) in GP2 as BB20s (Fig. 6b).

## Discussion

PRRSV has a high degree of genetic and antigenic variation [39]. It is known that PRRSV undergoes mutations, which sometimes result in the emergence of new PRRSV populations in PRRSV infected pigs [40–42]. During virus replication in vivo, the fittest virus variant distributions are sorted out by environmental parameters, and it is expected that the adaptive immune response of the host will act as an important source of selective pressure in the evolutionary process of the virus [33, 34]. In addition, it was shown that the exertion of antibody-mediated selective pressure onto a neutralizing epitope (aa 57–68) of GP4 of a European genotype PRRSV strain rapidly resulted in the selection of viable PRRSV variants that were resistant to neutralization by these antibodies and that carried aa substitutions in the epitope [36]. All these findings support the hypothesis that NAbs may be a driving force of the variability in the neutralizing epitope. And under the selective pressure, PRRSV will accumulate aa substitutions in the attachment proteins that select for resistance to neutralization by NAbs. In our previous study, it was confirmed that the aa mutants Y102C and G104R in GP5 played an important role in escaping from the neutralizing of HP-PRRSV NAbs. Meanwhile, the resistant variant BB20s, which has no aa mutants in GP5, significantly reduced the NA titers compared to parent virus BB [38]. In this study, 12 recombinant PRRSV strains containing these aa residue substitutions were constructed using reverse genetic techniques based on the sequence differences of the structural proteins between BB20s and the parent virus strain BB. It was firstly confirmed that the aa residues at 70 within M of PRRSV played an important role in regulating the neutralization susceptibility of PRRSV to NAbs.

GP2 interacts with GP3 and GP4 to form a multiprotein complex that is dispensable for viral particle formation, yet is important for viral infectivity [14, 43]. There are several lines of evidence for the existence of neutralizing epitopes in other minor GPs of PRRSV. Two linear antigenic regions, aa 37–48 and 117–128, have been identified within the minor envelope protein GP2 of the European genotype PRRSV strain Lelystad virus [44]. In this study, our results indicated that aa 198 within GP2 of the type 2 PRRSV strain BB was one antibody binding site, as determined using the recombinant virus rBB/GP2s. However, we did not obtain the same results when using rBB20s/GP2. The NA titer of rBB20s/GP2 was only slightly higher than that of rBB20s. Considering that GP2 is a minor component of the PRRSV envelope, the 198 aa in GP2 should be an antibody binding site in type 2 PRRSV strains. By using 217 peptides printed on a chip and reacted with anti-HP-PRRSV antibody, an antigen reaction active region (AR) from residue Y^51^ to S^106^ in GP3 was identified [45]. In addition, European genotype PRRSV strains strongly induce NAbs against the hypervariable region aa 57–68 of GP4 [19, 21, 22]. In this study, the aa substitutions in GP3 and GP4 did not exhibit the resistant ability against the NAb serum. The possible reason might be the NAb serum used in this study, and the role of GP3 and GP4 as targets for NAbs against type 2 PRRSV strains should be elucidated in the future.

NAbs to the M protein has also been reported, but the specific epitopes have not been identified [23, 31]. One report suggested that DNA shuffling of the GP4 or M genes from different parental viruses can broaden the cross-neutralizing antibody-inducing ability of the chimeric viruses against heterologous PRRSV strains. And sequence analyses of the shuffled viruses revealed that the arginine (R) mutation at position 70 in M could potentially be related to the enhanced cross-neutralizing antibody [46]. Here, the 70 aa in M protein was also mutated by passaging under the NAbs pressure. And the R70K substitution in M of the recombinant PRRSV rBB/Ms significantly reduced the N4 NA titer compared to the parent virus rBB. Meanwhile, comparison of the mutation rate between different PRRSV proteins and different regions within these proteins revealed that regions that are exposed at the PRRS virion surface, like the ectodomain of GP5, are much more variable than internal regions or proteins [40, 41]. The aa position of 70 in M protein was just located at the ectodomain region (aa 63–70) [10]. Ultimately, the consistency of these researches further confirmed that the 70 aa site in M was one antibody binding site, and the aa substitution at this position will regulate the neutralization susceptibility of PRRSV to NAbs. When we analyzed the nAb titer against PRRSV S1 strain with 70 K in the M protein, we found that the titer against S1 was significant lower than that against BB strain with 70R in the M protein (data not shown here). In addition, during the passage of PRRSV strain BB, we also used one negative anti-PRRSV serum as negative pressure to obtain the control strains. The sequence results revealed that the control virus at passage 20 does not have aa substitutions at 70 in M (data not shown here). Of course, further studies should be needed to address the importance of other putative antigenic sites using different anti-PRRSV serums.

The antigenic variation of PRRSV mitigates the effectiveness of virus recognition and subsequent immune responses. Although the utilization of PRRSV MLV is an effective method to provide immunological protection against homologous or genetically similar strains, the protection by the MLVs against the heterologous strains of PRRSV is limited [47–49]. In this study, the comparison of aa sequences of M proteins reveals that the aa residue 70 K also exists in the vaccine strain MLV. Considering the latest hypothesis that the arginine (R) mutation at position 70 in M could potentially be related to the enhanced cross-neutralizing antibody [46] and the antibody binding site confirmed in this study, there may be a relationship between the 70 K residue in M and the limit cross protection of the MLV. In addition, The rapid adaptation of PRRSV to an anti-viral environment, like neutralizing antibodies, indicates that PRRSV vaccines should focus on quickly and robustly inducing multiple arms of the immune system, as suggested before [36].

## Conclusions

In summary, in this study we demonstrated for the first time that the aa residue at 70 in M protein of PRRSV played important role in regulating neutralization susceptibility to the porcine serum NAbs. It may be helpful for monitoring the antigen variant strains in the field and developing new vaccine against PRRSV in the future.

## Methods

### Cells, viruses and NAb serums

MARC-145 cells were maintained in Dulbecco’s Modified Eagle’s medium (DMEM, GIBCO, Carlsbad, CA, USA) supplemented with 10 % fetal bovine serum (FBS, GIBCO) containing 100 U penicillin/ml and 100 μg streptomycin/ml at 37 °C with 5 % CO_2_. PAM cells were prepared from 4-week-old PRRSV-free piglets by lung lavage as previously described [38], and suspended with complete RPMI-1640 (GIBCO) medium, which contained 10 % fetal bovine serum, 100 units/mL of penicillin, and 100 μg/mL of streptomycin.

The HP-PRRSV isolate BB0907 (GenBank no. HQ315835, termed BB) used in this study was isolated in Guangxi Province, China, in 2009. The three resistant PRRSV variants BB5s, BB10s and BB20s, were obtained as previously described [38]. The recombinant PRRSV strains (rBB, rBB/GP2s, rBB/GP3s, rBB/GP4s, rBB/Ms, rBB/Ms-R, rBB20s, rBB20s/GP2, rBB20s/GP3, rBB20s/GP4, rBB20s/M and rBB20s/M-R) were constructed by site-directed mutagenesis of the aa residues according to conventional methods (Fig. 1). The titers of the viral stocks were determined by measuring their cytopathic effect (CPE) in MARC-145 cells.

Five different anti-HP-PRRSV neutralizing serums named N1, N2, N3, N4 and N5, were prepared from five 45-day-old piglets free of PRRSV by inoculated with low dose (10^4^ TCID_50_) of HP-PRRSV strain BB by 2 times with interval 28 days. The piglet blood samples were collected and the serums were isolated at 70 days post inoculation [38]. The NA titers of these serums against HP-PRRSV BB were 1: 34–1:48.

### Isolation of antibody-resistant variants

Three resistant variants BB5s, BB10s and BB20s against HP-PRRSV NAbs were obtained as previously described [38]. In brief, PRRSV strain BB (100 TCID_50_) was mixed with a 100-fold dilution of the antibody serum N4, which had the highest NA titer (1:48), and incubated at 37 °C for 1 h before infecting MARC-145 cell monolayers. The virus arising from the cells showing a CPE was used for the subsequent passage. After passaged by 5–20 times under the antibody pressure, three NAb-resistant variant clones (BB5s, BB10s and BB20s) were selected by using plaque tests. The titers of the resistant virus were 10^6^–10^8^ TCID_50_/ml.

### Serum neutralization assay on MARC-145

The NA titers of the antibody serums against the parent and mutant HP-PRRSVs on MARC-145 were assayed as described previously [50] with minor modifications. The viruses were diluted to a concentration of 100 TCID_50_ per 50 μl (10^3.3^ TCID_50_/ml) in DMEM supplemented with 5 % FBS. Serial dilutions of the neutralizing serums were mixed with each of the viruses and incubated at 37 °C for 1 h. The mixtures (100 μl/well) were transferred to MARC-145 monolayers in 96-well plates and incubated for an additional 2 days at 37 °C with 5 % CO_2_. The cells were fixed for 10 min with a solution of 50 % methanol and 50 % acetone. After washing with PBS, expression of the N protein of PRRSV was detected by mAb against the N protein (made in our laboratory) and FITC-conjugated goat anti-mouse IgG (BOSTER, China). The NA titers were determined as the reciprocal of the highest dilution that resulted in more than 90 % reduction of infected cells.

### Single replication cycle virus-neutralization test on porcine alveolar macrophages (PAMs)

The isolated PAM cells were added to 96-well culture flasks (Costar, Corning Incorporated, NY) and incubated for 6 h at 37 °C in a humidified compartment to allow cells to adhere to flasks. Single replication virus-neutralization test on PAMs was essentially performed as described [19] with minor modifications. Two-fold serial dilutions of serum N4 in RPMI-1640 were mixed with equal volumes of virus resulting in a final titre of 10^5^ TCID_50_/ml. RPMI-1640 without serum was included as mock condition. Virus-antibody mixtures were incubated for 1 h at 37 °C and transferred to a 96-well plate (100 μl/well) with PAMs. The inocula were removed after 1 h and replaced by medium, after which the cells were further incubated for another 10 h. The cells were fixed and stained with mAb against the N protein of PRRSV and FITC-conjugated goat anti-mouse IgG (BOSTER, China). The NA titers were determined as the reciprocal of the highest dilution that resulted in more than 90 % reduction of infected cells.

### Construction of infectious cDNA clones of PRRSVs

The full-length PRRSV genome was amplified using the five primer pairs listed in Additional file 1: Table S1. The recombinant plasmid pCMV-BB containing full-length cDNA of BB and pCMV-BB20s containing full-length cDNA of BB20s were constructed as shown in Fig. 1. To introduce the aa mutations of the structural proteins into PRRSV infectious cDNA clone pCMV-BB or pCMV-BB20s, site-directed mutagenesis was employed as described before [38]. All primers for the construction of site-directed mutations were listed in Additional file 1: Table S1. Besides, two different reverse mutants that were constructed from the full-length infectious cDNA clones pCMV-BB/Ms and pCMV-BB20s/M by the site-directed mutagenesis, respectively (Fig. 1). All the recombinant viruses and the aa mutations are summarized in Table 1.

### Rescue of recombinant viruses

Plasmids carrying full-length PRRSV cDNAs were individually transfected into MARC-145 cells using Lipofectamine 2000 (Invitrogen) according to the manufacturer’s instructions. Four days after transfection, the rescues of infectious viruses were obtained and cloned by the plaque assay. The mutations in the rescued viruses were confirmed by RT-PCR and sequencing.

### Viral plaque assay

MARC-145 cells in 12-well plates were inoculated with 100 μl of tenfold serially diluted PRRSV. After 1 h adsorption at 37 °C, cell monolayers were washed with phosphate-buffered saline (PBS) and overlaid with 1 % low melting agarose in DMEM (Invitrogen) containing 2 % FBS. After the gel overlay solidified, the plates were inverted (top side down) and placed into an incubator at 37 °C with 5 % CO_2_. At 4 days post-infection (dpi), plaques were visualized by crystal violet staining.

### Antibody binding analyses

Parent and mutant PRRSVs were purified by ultracentrifugation and diluted in coating buffer to a final concentration 5 μg/ml. The antigens were added in triplicate to 96-well flat-bottomed enzyme-linked immunosorbent assay (ELISA) plates. After incubation at 4 °C for at least 16 h, wells were blocked with PBS-5 % FBS for 1 h at room temperature. A non-saturating concentration of the serum antibody (diluted 1:100), which was in the linear portion of the antibody titration curve determined previously, was added to each well, serially diluted twofold, and incubated with the virus-coated plates for 1 h at room temperature. After washing, anti-pig horseradish peroxidase (HRP)-conjugated antibody was added to the plates, and they were incubated for 1 h at room temperature. After another round of washing, 3,3′,5,5′-tetramethylbenzidine (TMB) substrate (KPL Biomedical, Gaithersburg, MD) was added, and 2 M H_2_SO_4_ was used to stop the reaction. The amount of HRP product was determined using a plate reader at 450 nm. The control plates were coated with ultracentrifuged non-infected MARC-145 cell lysates.

### Western blot

In order to evaluate the consistence of PRRSV antigens binding in the wells, the antigens were lysed from the coated 96-well ELISA plate by using RIPA Lysis Buffer (Beyotime, China). Briefly, 100 μl lysis buffer was added into one coated well. After 5 minutes, the lysate samples were obtained and added to another coated well. A total of 12 wells (for one strain antigen) were lysed and obtained as one sample. Then all the PRRSV strains antigen samples from the coated plate were separated by 12 % SDS-PAGE, and transferred onto nitrocellulose filter membrane (PALL, New York, USA). The membranes were incubated with mAb N (made in our laboratory) as the primary antibody. After the membranes were rinsed with PBS, the membrane was treated with goat anti-mouse IgG-HRP (BOSTER, China) as the secondary antibody. The proteins were visualized by scanning the membranes with the Tanon 5200 chemiluminescence imaging system (Tanon, China).

### Growth curves of viruses

To determine viral one-step growth curves, all PRRSV mutants (10^5^ TCID_50_) were inoculated into sub-confluent MARC-145 cells or PAMs in six-well plates. Then, 100 μl of the supernatants of the infected cells was collected and replenished with the same volume of fresh medium at 12, 24, 36, 48, and 72 h post-infection(hpi), and stored at −70 °C for virus titration. The virus titers for each time point were determined by TCID_50_.

### Statistical analysis

All data were analyzed using GraphPad Prism (Version 5.03, San Diego, California) software. Differences among all groups were examined using one-way analysis of variance (ANOVA), followed by Tukey’s tests. Differences between two groups were assessed using unpaired two-tailed *t*-tests. Differences were considered significant if *P* was <0.05.

### Ethics statements

All animal protocols were approved by the Animal Care and Ethics Committee of Nanjing Agricultural University (permit number: IACECNAU 20121001) and followed the Guiding Principles for Biomedical Research Involving Animals.

## Additional file

Additional file 1: Table S1. Primer sequences for construction of the subgenomic replicon of PRRSV and site-directed mutagenesis. (DOC 54 kb)
