# Supplementary material for: Influence of the amino acid residues at 70 in M protein of porcine reproductive and respiratory syndrome virus on viral neutralization susceptibility to the serum antibody
Source: Virol J. 2016 Mar 22;13:51. doi: 10.1186/s12985-016-0505-7 (PMC4802621; doi:10.1186/s12985-016-0505-7)
Supplement: Additional file 1: Table S1. — Primer sequences for construction of the subgenomic replicon of PRRSV and site-directed mutagenesis. (DOC 54 kb) [file 12985_2016_505_MOESM1_ESM.doc]

**Table S1.** Primer sequences for construction of the subgenomic replicon of PRRSV and site-directed mutagenesis.

| Primers | Sequence (5’-3’) | Purpose |
| --- | --- | --- |
| Primers for the construction of infectious, full-length cDNA clones of BF10 | |  |
| A-1-F | GCGTTAATTAAACCGTCATGACGTATAGGTGTTG | Fragment A |
| A-3408-R | TGTCTCGAGAATCATCTTTGGGAGAAACC | Fragment A |
| B-3383-F | TTCTTAATTAAATGATTCTCGAGACACCGCC | Fragment B |
| B-6473-R | GTGCTTAAGTTCATTACCACCTGTAACGGAT | Fragment B |
| C1-6448-F | GCGTTAATTAAAATGAACTTAAGCACCTATGCC | Fragment C  (SOE PCR) |
| C1-8979-R | TTGACACAGAGGTAATCGGGTCGCCAGAC |
| C2-8951-F | GTCTGGCGACCCGATTACCTCTGTGTCAA |
| C2-11945-R | CGGGGGAAAATGAAACCTCATGCTGGT |
| D-11865-F | TCGTTAATTAAGTTTCGGGCGCGCCAGAAAGGG | Fragment D |
| D-15425(SwaI)-R | **TTCGGCTTGGGATTT**AAATATGCATTTTTTTTTTTTTTTTTTTTT | Insert poly A tial |
| D-15464(SpeI)-R | CTCACTAGTAACGGCCGCCAGTGTGCTGGAA**TTCGGCTTGGGATTT** | Insert SpeI |
| Primers for the construction of site-directed mutations in plasmid pCMV-BB | |  |
| GP2(198G/V) F | TTCCCAACCCCTGTTTCCCGGCCAAA | Site-  directed mutagenesis |
| GP2(198G/V) R | TTTGGCCGGGAAACAGGGGTTGGGAA |
| GP3(69P/S,71K/R)F | CTGAGACCCTTGAATCCGGCAGGTCTTTTTGGTG |
| GP3(69P/S,71K/R)R | CACCAAAAAGACCTGCCGGATTCAAGGGTCTCAG |
| GP3(226S/P)F | CCGCAGCATCAGACTCCATTGTCCTCCAG |
| GP3(226S/P)R | CTGGAGGACAATGGAGTCTGATGCTGCGG |
| GP4(43D/N;44F/S)F | CCACCGCAGCATCAAACTCCATTGTCCTCCAGGA |
| GP4(43D/N;44F/S)R | TCCTGGAGGACAATGGAGTTTGATGCTGCGGTGG |
| M(70R/K)F | TGAGAGCACAAATAAGGTCGCGCTCACT |
| M(70R/K)R | AGTGAGCGCGACCTTATTTGTGCTCTCA |
| Primers for the construction of site-directed mutations in plasmid pCMV-BB20s | | |
| GP2(198V/G) F | TTCCCAACCCCTGGTTCCCGGCCAAA | Site-  directed mutagenesis |
| GP2(198V/G) R | TTTGGCCGGGAACCAGGGGTTGGGAA |
| GP3(69S/P,71R/K)F | GAGACCCTTGAACCCGGCAAGTCTTTTTGGTGCA |
| GP3(69S/P,71R/K)R | TGCACCAAAAAGACTTGCCGGGTTCAAGGGTCTC |
| GP3(226P/S)F | CCGCAGCATCAGACTTCATTGTCCTCCAG |
| GP3(226P/S)R | CTGGAGGACAATGAAGTCTGATGCTGCGG |
| GP4(43N/D;44S/F)F | CCACCGCAGCATCAGACTTCATTGTCCTCCAGG |
| GP4(43N/D;44S/F)R | CCTGGAGGACAATGAAGTCTGATGCTGCGGTGG |
| M(70K/R)F | TGAGAGCACAAATAGGGTCGCGCTCACT |
| M(70K/R)R | AGTGAGCGCGACCCTATTTGTGCTCTCA |
